# mARC vs. IMRT radiotherapy of the prostate with flat and flattening-filter-free beam energies

**DOI:** 10.1186/s13014-014-0250-2

**Published:** 2014-11-26

**Authors:** Yvonne Dzierma, Katharina Bell, Jan Palm, Frank Nuesken, Norbert Licht, Christian Rübe

**Affiliations:** Department of Radiotherapy, Saarland University Medical Center, Kirrberger Str. Geb. 6.5, 66421 Homburg, Germany

**Keywords:** mARC rotational treatment, Flattening-filter-free photons, Prostate cancer, Planning study

## Abstract

**Background:**

There as yet exists no systematic planning study investigating the novel mARC rotational radiotherapy technique, which is conceptually different from VMAT. We therefore present a planning study for prostate cancer, comparing mARC with IMRT treatment at the same linear accelerator equipped with flat and flattening-filter-free (FFF) photon energies.

**Methods:**

We retrospectively re-contoured and re-planned treatment plans for 10 consecutive prostate cancer patients. Plans were created for a Siemens Artiste linear accelerator with flat 6 MV and FFF 7 MV photons, using the Prowess Panther treatment planning system. mARC and IMRT plans were compared with each other considering indices for plan quality and dose to organs at risk. All plans were exported to the machine and irradiated while measuring scattered dose by thermoluminescent dosimeters placed on an anthropomorphic phantom. Treatment times were also measured and compared.

**Results:**

All plans were found acceptable for treatment. There was no marked preference for either technique or energy from the point of view of target coverage and dose to organs at risk. Scattered dose was significantly decreased by the use of FFF energies. While mARC and IMRT plans were of very similar overall quality, treatment time could be markedly decreased both by the use of mARC and FFF energy.

**Conclusions:**

Highly conformal treatment plans could be created both by the use of flat 6 MV and FFF 7 MV energy, using IMRT or mARC. For all practical purposes, the FFF 7 MV energy and mARC plans are acceptable for treatment, a combination of both allowing a drastic reduction in treatment time from over 5 minutes to about half this value.

## Background

The mARC (“modulated arc”) technique has recently been introduced as a rotational intensity-modulated radiation therapy (IMRT) technique for Siemens linear accelerators [1,2]. Although the dosimetric accuracy has been assessed by various methods and first patient treatment has been reported [3,4], no systematic planning studies have yet been carried out to assess the quality of mARC treatment as compared with IMRT delivered at the same linear accelerator.

First applications have centered on prostate treatment for mARC [4,5], which appears to be an ideal indication as it benefits from inverse planning due to the proximity of organs at risk (OAR), yet only requires one gantry rotation to achieve a highly conformal dose distribution (compare, e.g., [6]). We therefore present a planning study for prostate cancer with mARC for a Siemens Artiste machine equipped with flat 6 MV and flattening-filter-free (FFF) 7 MV energies. The combination of FFF beams with mARC treatment is of particular interest since this offers the greatest potential for a reduction in treatment time.

Although the mARC technique is a Siemens Artiste specific modality and primarily interesting for Siemens customers, we hope that the comparison of the flat and FFF beam lines, both for mARC and IMRT treatment, will be useful for a wide range of readers.

## Patients and methods

For the present study we chose 10 consecutive patients diagnosed with intermediate and high-grade prostate cancer that had previously been treated in our department. Due to the retrospective nature of this study, no ethics board approval was required. Patient characteristics are shown in Table 1. Computed tomography (CT) datasets had been acquired on a dedicated scanner (Brilliance CT - Big Bore Oncology, Philips, Koninklijke) with 3 mm spacing between slides. For all patients additional MRI data of the small pelvis was present for coregistration. For reasons of standardization contouring was completely redone by one radiation oncologist according to the Radiation Therapy Oncology Group (RTOG) Trial 0126 [7] contouring guidelines. Gross tumor volume (GTV), clinical target volume (CTV), planning target volume (PTV) and normal tissues were outlined on all CT slices in which the structures existed.

**Table 1:** **Patient characteristics**

	**Mean**	**Range**
**Age (years)**	69	59-74
**T-stage**		
**2a**	1	-
**2b**	1	-
**2c**	7	-
**3a**	1	-
**Gleason grading at diagnosis**	7	6-9
**PSA at diagnosis (ng/ml)**	26.56	3.8-94
**Anti androgen deprivation (yes/no)**	10/0	
**Prostate volume (ccm)**	61.6	39.55-91.22

The GTV encompassed the prostate gland, the CTV encompassed the GTV plus the proximal bilateral seminal vesicles (only the first 1.0 cm of the seminal vesicle tissue adjacent to the prostate). The PTV was generated by adding a surrounding margin of 7 mm to the CTV. Organs at risk were outlined according to the Male RTOG Normal Pelvis Atlas [8]. A dose of 76 Gy was prescribed to the PTV, for plan evaluation we used our in-house DVH (dose-volume histogram) criteria as shown in Table 2 that are mainly based on the data published by Quantitative Analysis Of Normal Tissue Effects In The Clinic (QUANTEC) [9,10].

**Table 2:** **DVH criteria for plan acceptability**

PTV	V(95%) >95%
	V(105%) <5%
Bladder	V75Gy <15%
	V70Gy <20%
	V50Gy <50%
Rectum	V70Gy <10%
	V60Gy <30%
	V50Gy <50%
	V40Gy <70%
	V30Gy <80%
Posterior rectal wall	D(max) <60 Gy
	V50Gy <15%
	V40Gy <30%
Femoral heads	V50Gy <5%

At our institution, the mARC technique is available at one Siemens Artiste linear accelerator with flat 6 MV and FFF 7 MV energy, equipped with 160 multi-leaf collimator (MLC, leaf width 5 mm). The two energies are particularly well suited for comparative planning as the slightly increased nominal energy of the FFF 7 MV beam compensates for the spectral softening caused by the removal of the flattening filter, so that the percent depth dose of the FFF 7 MV beam matches the flat 6 MV beam closely [11]. Therefore, any differences in plan quality will reflect mainly the difference in beam profiles.

For mARC plans, one complete (360°) gantry rotation was used with optimization points spaced 10° and arclet length 4°. IMRT plans consisted of 11 beams (gantry angles 205°, 235°, 265°, 295°, 330°, 0°, 30°, 65°, 95°, 125°, 155°), with 3 segments per beam. In a prior test, it was checked if plans were improved by allowing 5 segments per beam (the “gold standard” for prostate IMRT at our institution). Since no significant difference was observed, we here limit our analysis to IMRT plans with a total of 33 segments or less, which is nearly the same number of degrees of freedom as for the mARC plans (36 optimization points). The collimator angle was 90° for all plans, also based on previous tests.

Planning is performed in the Prowess Panther V5.10r2 treatment planning system (TPS) on a 3 mm dose grid using the collapsed cone dose algorithm. IMRT and mARC inversion are closely similar, both using a simulated annealing approach for direct aperture optimization. Based on a set of inversion objectives, the optimization can be carried out interactively by adjusting the DVH constraints and weights until the desired shape is reached. Criteria for optimization are listed in Table 2.

Plan quality was compared for the four scenarios (IMRT vs. mARC, 6 MV vs. FFF 7 MV) based on the conformity index (CI), the homogeneity index (HI), V(50Gy) for bladder and rectum, and V(40Gy) of the posterior rectal wall. The conformity index is defined by [12]:

$$ CI=\frac{T{V}_{PIV}{}^2}{TV\cdot PIV} $$

where *TV* denotes the volume of the PTV*, PIV* is the volume enclosed in the prescribed isodose (95%), and *TV*_*PIV*_ is the volume of the PTV surrounded by the prescribed isodose (95%).

The homogeneity index is calculated as

$$ HI=\frac{D_{PTV}\left(2\%\right)-{D}_{PTV}\left(98\%\right)}{D_{PTV}\left(50\%\right)} $$

where *D*_*PTV*_*(x %)* means the dose received by x % of the PTV volume.

All plans were exported to the machine for treatment and irradiated on an Alderson anthropomorphic phantom positioned with the prostate at the approximate location of the isocenter. Thermo-luminescent dosimeters (Harshaw TLD 100H) were placed at three positions on the surface of the phantom (navel, manubrium sterni, right eye lens) to measure the scattered dose outside the treatment field. At each position, three TLDs were placed in close proximity and the measurements averaged. The average standard deviation of the three measurements was below 5% for the measurements at the navel, and between 5 and 10% for the lower dose values measured at the sternum and lens. During irradiation, treatment times were measured for comparison.

Plans were compared pair-wise for mARC vs. IMRT and for 6 MV vs. 7 MV energy, considering the measures of quality defined above, monitor units, treatment time and scattered dose. The Shapiro-Wilk test was performed to check for normality. In cases where this could not be refuted, the t-test for paired data was applied, otherwise the Wilcoxon signed-rank test was used. A value of p = 0.05 or below was considered to be statistically significant.

## Results

All plans (DVH and dose distributions) were reviewed by at least one senior radiotherapist and were all deemed acceptable for treatment. All plans satisfied the criteria that at least 95% of the planning target volume received 95% of the prescribed dose of 76 Gy, and all organs at risk remained below the imposed limits (Table 2).

**Figure 1:**
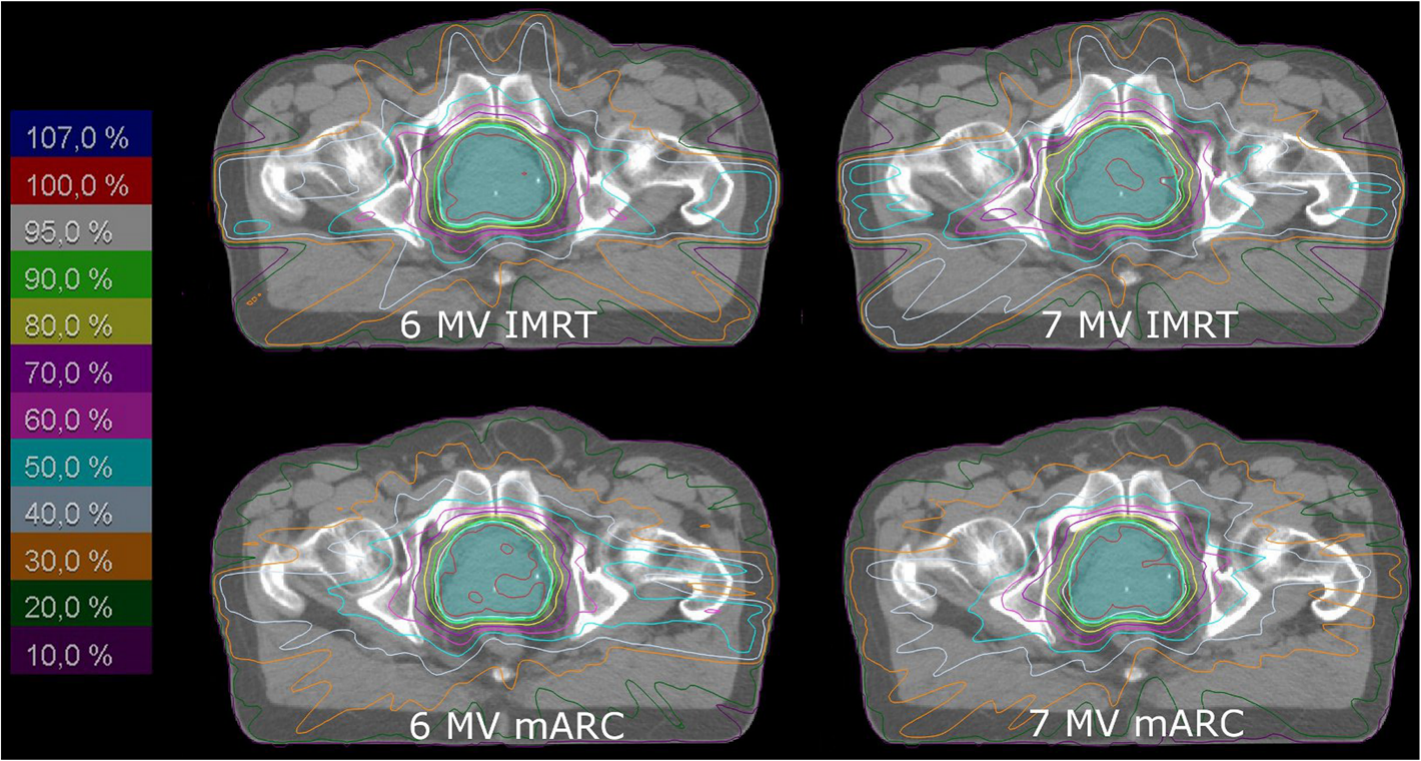
**Example dose distributions (transverse slices).** The isodose lines are given relative to the reference point, which receives 76 Gy. The PTV is displayed as filled cyan contour.

**Figure 2:**
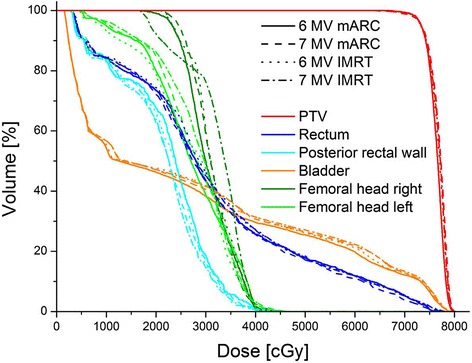
**Example dose-volume histogram (same patient as in** **Figure** 1 **).**

A visual comparison of the four plan scenarios for each patient did not show a marked preference for either technique or energy (example dose distributions and DVH shown in Figures 1 and 2). DVHs of the four scenarios are closely similar for each patient. Relying on the quality measures (Table 3), the comparison of IMRT with mARC plans for the same energy never yielded a significant difference. For comparison of 6 MV with FFF 7 MV, homogeneity index and conformity index were significantly better for 6 MV than 7 MV both for mARC and IMRT plans, separately. Considering dose to OAR, there was no significance for comparison of IMRT plans (6 MV vs. 7 MV), but 6 MV mARC plans performed better than 7 MV mARC. However, even in those cases where a statistical significance was shown, the differences were very small and hardly of clinical significance: For the bladder, a median of 20.8% of the volume received a dose of 50 Gy in the 6 MV mARC plans, whereas this was 22.4% for 7 MV mARC. The rectum V50 increases from 16.8% for 6 MV mARC to 17.5% for 7 MV mARC. As all these values remain far below the allowed limits, they would not have caused rejection of the plans for clinical treatment. For all these values, the variation between different patients was considerably larger than the variation from one plan scenario to the next, which can be seen by the overlapping ranges of values (Table 3) even in cases where a small statistical significance was found.

**Table 3:** **Measures of quality, monitor units, treatment times and scattered dose for the four plan scenarios (median values ± standard deviation (minimum-maximum), and p-values of pair-wise tests)**

**Index**	**6 MV IMRT**	**7 MV IMRT**	**6 MV mARC**	**7 MV mARC**	**p (6 MV IMRT – mARC)**	**p (7 MV IMRT – mARC)**	**p (IMRT 6 MV – 7 MV)**	**p (mARC 6 MV – 7 MV)**
CI	0.896 ± 0.024 (0.855-0.945)	0.883 ± 0.014 (0.864-0.910)	0.909 ± 0.017 (0.878-0.925)	0.883 ± 0.024 (0.850-0.926)	n.s.	n.s.	0.029	0.004
HI	0.096 ± 0.010 (0.080-0.111)	0.100 ± 0.010 (0.093-0.122)	0.091 ± 0.011 (0.085-0.121)	0.102 ± 0.010 (0.088-0.126)	n.s.	n.s.	0.010	0.027
Bladder V50Gy (%)	21.0 ± 14.0 (6.3-48.7)	22.1 ± 14.1 (6.3-48.2)	20.8 ± 14.2 (6.0-49.3)	22.4 ± 14.6 (6.7-49.8)	n.s.	n.s.	n.s.	0.004
Rectum V50Gy (%)	16.9 ± 5.1 (5.9-22.2)	17.2 ± 5.2 (7.1-23.7)	16.8 ± 5.2 (5.3-22.3)	17.5 ± 5.5 (5.7-23.4)	n.s.	n.s.	n.s.	0.049
Post. Rect. Wall V40Gy (%)	3.8 ± 3.4 (0.0-9.7)	3.6 ± 4.2 (0.0-12.7)	3.5 ± 3.2 (0.0-8.3)	4.8 ± 4.2 (0.0-11.6)	n.s.	n.s.	n.s.	0.043
MU	402 ± 29 (365–442)	402 ± 25 (368–445)	414 ± 29 (365–452)	411 ± 43 (366–507)	0.047	n.s.	n.s.	n.s.
Treatment time (min:sec)	5:21 ± 0:10 (5:09–5:40)	4:31 ± 0:04 (4:25–4:39)	3:35 ± 0:12 (3:20–3:55)	2:27 ± 0:09 (2:13–2:39)	0.002	0.002	0.002	0.002
Dose at navel (mGy)	15.5 ± 2.1 (12.8-18.8)	12.2 ± 1.7 (10.8-15.3)	14.6 ± 2.3 (12.7-19.0)	12.4 ± 2.2 (10.3-16.7)	n.s.	n.s.	0.002	0.002
Dose at manubrium (mGy)	1.47 ± 0.2 (1.21-1.86)	1.25 ± 0.2 (1.06-1.59)	1.52 ± 0.2 (1.33-1.87)	1.29 ± 0.2 (0.97-1.58)	n.s.	n.s.	0.002	0.002
Dose at lens (mGy)	0.597 ± 0.046 (0.534-0.689)	0.351 ± 0.030 (0.320-0.423)	0.597 ± 0.050 (0.563-0.705)	0.378 ± 0.037 (0.322-0.449)	n.s.	0.020	0.002	0.002

n.s.: not significant.

The mARC plans required slightly more monitor units than the IMRT plans, but the difference was negligible (not significant except for 6 MV IMRT vs. mARC, with 402 and 414 MU, respectively, p = 0.047). However, treatment time could be markedly decreased both by the use of mARC and FFF energy. In moving from 6 MV to FFF 7 MV, about one minute treatment time was saved both in IMRT and mARC, respectively. In moving from IMRT (11 beams) to mARC, treatment times were reduced by about two minutes both for 6 MV and FFF 7 MV, respectively. By combining mARC treatment with FFF 7 MV energy, the treatment time could effectively be reduced by half (median 2:27 min for FFF mARC versus 5:21 min for 6 MV IMRT).

Scattered dose is significantly decreased by the use of FFF energies, which is physically reasonable since head scatter is reduced in the absence of a flattening filter. For identical plan scenarios, the FFF 7 MV energy produces only about 58–85% of the scattered dose measured for flat 6 MV, with strongest reduction at larger distance from the treatment field (lens). A difference between IMRT and mARC plans cannot be observed for 6 MV; for 7 MV, the out-of-field dose is slightly higher for mARC (up to 108% of the 7 MV IMRT plan, but still much lower than for the 6 MV plans).

## Discussion

### Monitor units

In this study, monitor units were not observed to differ significantly between mARC and IMRT, and for 6 MV vs. FFF 7 MV, respectively. This changes markedly if the isocenter is displaced from the centre of the PTV. In this case, the monitor units required by the 6 MV plans do not change systematically – sometimes increasing, sometimes decreasing, but remaining within 30 MU of the original value. For the FFF 7 MV plans, however, the value increases strongly, sometimes exceeding 500 MU. This is plausible, because if the isocenter moves to the side or even outside of the PTV, the dose intensity decreases with distance from the central axis, creating a constant dose gradient in the target. Additional monitor units are hence required to add dose at greater distance from the axis. This effect does not occur for the flat intensity profile of the 6 MV beam. If the isocenter is placed in the centre of the PTV, the dose profile of the FFF 7 MV beam peaks inside the PTV and only deviates from a flat profile at distances of several centimetres from the axis. It appears that the prostate PTV is sufficiently small to exhibit no notable difference in monitor units between 6 MV and 7 MV plans if the isocenter is centrally placed. If the PTV extended farther in the craniocaudal direction, it might be expected that the dose fall-off to the sides of the central axis – although symmetrical – would also require more monitor units for the FFF beam: this was indeed observed for large PTV [13,14]. Therefore, the position of the isocenter is more critical for the FFF energies.

### Treatment times

**Figure 3:**
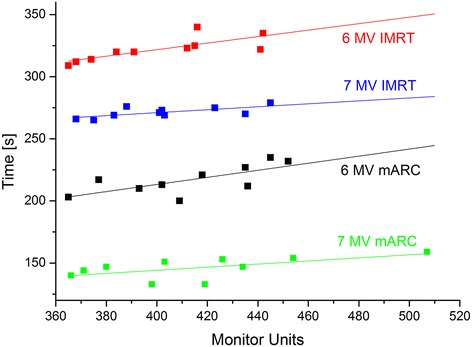
**Treatment time as a function of monitor units for the ten patients, with linear fits.** Fit parameters are given in Table 4.

Treatment times depend on the time for gantry and MLC movement on the one hand and on the time required to irradiate the monitor units on the other hand. As it is more time-consuming to stop the gantry at precise angles rather than just move it through an angular range, mARC saves treatment time in comparison with step-and-shoot plans that would use the same number of gantry angles (i.e., 36 with one segment per beam). The use of FFF beam energies saves time as the higher dose rate allows faster irradiation of the ca. 400 MU. We therefore assess the dependence of treatment time on the number of MU for the four scenarios (Figure 3). In all cases, a linear fit can be made, with parameters given in Table 4. Based on the above considerations, the y-axis intercept should be the same for both IMRT plans and for both mARC plans, respectively, since it is mainly determined by the irradiation geometry. The slope of the curves should be similar for the 6 MV plans and the 7 MV plans, respectively, since it depends on the available dose rate.

**Table 4:** **Linear fit parameters for treatment time vs. monitor units (Figure** **2** **)**

**Plan**	**y-axis intercept (s)**		**Slope (s/MU)**	
	**Value**	**Standard error**	**Value**	**Standard error**
6 MV IMRT	216.7	30.5	0.263	0.076
7 MV IMRT	233.5	18.3	0.119	0.045
6 MV mARC	98.7	41.1	0.286	0.099
7 MV mARC	94.1	23.9	0.125	0.057

Indeed, the y-axis intercepts of the IMRT plans for different energies differ less than one standard error; the same applies to the mARC plans. The slope of the curves for 6 MV plans also agree within less than one standard error, with an approximate value of 0.27 s/MU – this corresponds to an average dose rate of 219 MU/min. For the 7 MV plans, the slope (again within less than one standard error) of ca. 0.122 s/MU corresponds to an average dose rate of 492 MU/min. These values are reasonable – the maximum available dose rate for 6 MV is 300 MU/min. For FFF 7 MV, 2000 MU/min are theoretically available. However, for small segments/arclets with low MU, the linac firmware automatically reduces the dose rate for better linearity (for about 400 MU distributed over 33 segments or 36 arclets, the linac will nearly always operate at a reduced dose rate since most segments receive only about 10 MU). We therefore find that both the FFF IMRT and mARC plans operate at an average dose rate considerably below the maximum available; but still about twice as fast as the flat energy.

Considering the treatment time, the question arises what the technical limit for the mARC operation may be. Given the results above, it might be imagined that treatment times could be further improved if the plans were irradiated with the maximum available dose rates (300 MU/min for 6 MV and 2000 MU/min for FFF 7 MV). In addition, the spacing of optimization points and distance of MLC leaf travel between successive arclets will influence treatment time. Current work at our institution is aiming to find the optimum mARC scenarios for fast irradiation, and the technical constraints imposed on treatment time (Dzierma et al., in prep.). From the technical/legal point of view, gantry rotation is restrained to be no faster than 360° per minute, but only plan scenarios with hardly any MLC movement and few monitor units per arclet appear to be capable of achieving this speed. Future work will show where the practical limits for treatment times are, and treatment planning systems will then be evaluated by how closely they can approach this technical limit.

### Comparison with other studies

Only few studies have investigated the plan properties and treatment times associated with mARC planning [1,4,5]. Their observed treatment times for prostate cancer are of the same order of magnitude as those reported here, again for comparable plan qualities between mARC and IMRT treatments.

Several past studies have evaluated plan quality for VMAT/RapidArc treatment as compared with IMRT (for an overview, see [15]). While details differ between these studies – due on the one hand to variations in the considered prostate PTV contours, and on the other hand to different planning approaches – it was generally found that VMAT treatment offers at least as good quality plans as IMRT treatment, sometimes even with better sparing of organs at risk. Depending on whether one or two arcs were chosen for VMAT treatment and whether constant or variable dose rate irradiation was allowed, plan quality measures and OAR DVH values sometimes favoured IMRT, sometimes VMAT treatment [16-24]; however, all studies observed a marked reduction in treatment time by VMAT treatment, and generally a drastic reduction in monitor units.

Our study explicitly created the scenarios in such a way to offer approximately the same number of degrees of freedom to both IMRT and mARC optimization processes, which may explain the similar quality outcome. Besides, it should be pointed out that the studies comparing VMAT with IMRT all relied on IMRT plans with fewer fields – most chose 5 or 7 gantry angles for the IMRT, so it can be expected that our IMRT plans with 11 beams might yield better plans, hence closing the gap to rotational modulated treatment. Still, it has been observed that the plan quality can be improved by including more gantry angles even for the same number of segments [25], which is not surprising since it offers more freedom to the optimization from a geometrical point of view and might allow for better mARC plan quality even with a similar number of free parameters for the optimization. However, it also appears plausible that this effect should saturate for plans with many gantry angles – the more beams are used, the smaller would be the improvement by any additional beam, with an optimum number of ca. 10–20 beams depending on the target size [26]. In fact, our in-house standards have evolved over the past few years from IMRT plans with 5–7 beams to 11–13 beams for prostate cancer. More beams are never used since they have not shown to yield any further benefit, which may explain why the mARC plan quality is not notably improved over the IMRT plans.

Considering the monitor units, we do not observe a marked decrease for mARC vs. IMRT plans, whereas most studies considering VMAT vs. IMRT treatment do. The monitor units for the mARC plans are comparable to those found by, e.g., [6,16,19-21,23,24,27] all of whom except for Ost et al. [27] had considerably higher IMRT monitor units. It cannot be decided whether the low amount of MU also for IMRT is indebted to the planning scenario (more degrees of freedom offered by 11 beam angles) or the planning system (the direct aperture optimization algorithm used in Prowess Panther has been observed to require fewer MUs for IMRT in comparison with other planning systems [28]). The low number of monitor units for our IMRT plans also entails relatively fast IMRT treatment (5:21 minutes for flat and 4:31 minutes for FFF beams), which is at the lower limit of what is observed in other studies (4–6 min [6,17,19,20,22,23,27,29,30]; 8 min [21,31]). mARC treatment times of 3:35 min for flat and 2:27 min for FFF treatment plans are slower than times reported for VMAT treatment with single arcs (1–2 minutes [6,17,19-21,27]; FFF: 60–90 sec [14,30,32]), but within the range of times found for two arcs (3–5 min [6,20,21,23]).

## Conclusion

Although small differences in plan quality exist, none of these were found to be clinically significant. Highly conformal treatment plans could be created both by the use of flat 6 MV and FFF 7 MV energy, using IMRT or mARC. For all practical purposes, the FFF 7 MV energy and mARC plans are acceptable for treatment, allowing a drastic reduction in treatment time from over 5 minutes to about half this value. As expected on physical grounds and based on past studies, scattered dose is reduced by the FFF 7 MV energy.

### Consent

Written informed consent was obtained from the patient for the publication of this report and any accompanying images.
